# Leaky Gut, Leaky Brain?

**DOI:** 10.3390/microorganisms6040107

**Published:** 2018-10-18

**Authors:** Mark E. M. Obrenovich

**Affiliations:** 1 Research Service, Louis Stokes Cleveland Department of Veteran’s Affairs Medical Center, Cleveland, OH 44106, USA; MEO5@CASE.EDU; 2 Department of Chemistry, Case Western Reserve University, Cleveland, OH 44106, USA; 3 The Gilgamesh Foundation for Medical Science and Research, Cleveland, OH 44116, USA; 4 Department of Medicinal and Biological Chemistry, College of Pharmacy and Pharmaceutical Sciences, University of Toledo, Toledo, OH 43606, USA; 5 Departments of Chemistry and Biological and Environmental Sciences, Cleveland State University, Cleveland, OH 44115, USA

**Keywords:** leaky gut, leaky brain, microbiota, microbiome, celiac disease, gluten, gluten-free, microbiota-gut-brain axis, metabolic interactome, inflammation, blood barriers

## Abstract

‘Leaky gut’ syndrome, long-associated with celiac disease, has attracted much attention in recent years and for decades, was widely known in complementary/alternative medicine circles. It is often described as an increase in the permeability of the intestinal mucosa, which could allow bacteria, toxic digestive metabolites, bacterial toxins, and small molecules to ‘leak’ into the bloodstream. Nervous system involvement with celiac disease is know to occur even at subclinical levels. Gluten and gluten sensitivity are considered to trigger this syndrome in individuals genetically predisposed to celiac disease. However, the incidence of celiac disease in the general population is quite low. Nevertheless, increased public interest in gluten sensitivity has contributed to expanded food labels stating ‘gluten-free’ and the proliferation of gluten-free products, which further drives gluten-free lifestyle changes by individuals without frank celiac disease. Moreover, systemic inflammation is associated with celiac disease, depression, and psychiatric comorbidities. This mini-review focuses on the possible neurophysiological basis of leaky gut; leaky brain disease; and the microbiota’s contribution to inflammation, gastrointestinal, and blood-brain barrier integrity, in order to build a case for possible mechanisms that could foster further ‘leaky’ syndromes. We ask whether a gluten-free diet is important for anyone or only those with celiac disease.

## 1. Introduction

The mutually beneficial relationship between the host and its resident gut microbiota has been described by many [1,2,3]. Bacterial products and metabolites from gut commensal micro-organisms are largely useful for the host and our overall health. Arguably, the most important of these is co-metabolism, which occurs between the microbiota and host systems, and the same microbes can control integral segments of our neurobiology and even affect several mammalian systems like the brain and digestive system [2]. The microbiota-gut is an integral component of the gut–brain neuroendocrine metabolic axis [2] and any disruption that can occur, such as antibiotic use and during disease, could upset homeostasis and share an inflammatory component [2]. Celiac disease, ulcerative colitis, or Crohn’s disease—the latter two are collectively referred to as inflammatory bowel disease—are chronic conditions that affect the gastrointestinal tract and have an inflammatory component. The microbiota gastrointestinal barrier, together with transport proteins, act at the interface of blood permeability barriers to help regulate trafficking of macromolecules between the digestive environment and the host. How diet, lifestyle, antibiotics, and other factors shape the microbiota-gut, and its integrity, constitutes a rapidly growing area of research interest.

Commensal bacteria, together with the gut-associated lymphoid tissues like pyre’s patches and the neuroendocrine network, form a close relationship between the digestive system and neural or cognitive functions. What is newer involves global changes like neuro-inflammation and excitatory states, as in celiac disease, which can affect distal organs including the brain. It is here that the gut can influence the blood brain barrier (BBB) by means of gastrointestinal-derived hormonal secretion, small molecule and metabolic cofactor production, or through cytokine stress and other inflammatory mechanisms to even affect blood-brain barrier permeability [4]. We find that autistic spectrum disorder (ASD) and schizophrenia share some features with celiac disease, including production of microbial-derived compounds found in brain compartments [3,5,6]. Further, the brain coordinates chemokine-regulated cellular recruitment releases cytokines and mediates cellular removal of pathogenic material and microbial compounds to avoid brain injury [6]. The gut microbiome regulates innate immunity though peripheral immune cell populations and modulates microglial phenotype [3,6]. Perturbations in the microbiota can induce alterations in pathological phenotypes associated with neurodegeneration [2,6]. Regulating neuroinflammation via the host microbiota highlights the potential importance of the BBB and blood–cerebrospinal fluid (CSF) barrier. Main and colleagues describe these blood-barriers as adjunct immune barriers that communicate host immune status to the brain [6].

The expanded concept of a leaky gut suggests that with age, and under specific conditions, small molecule bacterial metabolic components can translocate or diffuse systemically, and even disseminate to distal sites from the gut epithelial barrier to even pass the blood brain barrier and reach the CSF or other brain compartments [1,2,3,6,7,8]. For example, toxin A and toxin B from Clostridium difficile are both implicated in the pathogenesis of pseudomembranous colitis [9], without bacterial translocation or systemic dissemination. This is a new way of viewing the so-called leaky gut. Here, these bacterial metabolites, or toxins, eventually may both contribute to disease or modulate health biochemically and immunologically by both direct and indirect means without causing sepsis or infection. Through mass action, for instance, metabolic pathways could be masked, inhibited, or fed forward by increases in select pathway precursors or stressors and propagate intermediates to eventually increase deleterious end-product formation or via general inflammation [1,10].

However, what is not well characterized is co-metabolism within the concept of a “leaky gut” and in the movement of bacterial metabolites, which affects these and other gut processes. The net metabolic cross-talk occurring with co-metabolism it is staggering, so it is not surprising that the human central nervous system (CNS) is under constant assault or, conversely, does benefit from a wide mix of extrinsic and intrinsic neuro-psychotropic-modulating microbes, pathogens, and their metabolites. It is important to to remember the gut microbiota and the brain are not necessarily in opposition to one another, but work together for proper functioning of the CNS, which may be dependent upon the presence of correctly balanced microbial populations. As the CNS has evolved and developed in the presence of the microbiota, dysfunction is as likely to arise from the absence or disruption in normal microbial components as it is from their inappropriate distribution ratios. In addition to bacteria, other pathogens make understanding the net metabolic cross-talk formidable and include, among other compounds, environmental toxins and microbial-derived small non-coding RNAs, viruses, and fungi to name a few [1].

Critical to any discussion on leaky barrier systems are pathogens, which, unlike commensals, have evolved elaborated mechanisms to target host barrier integrity and disseminate systemically to invade deeper tissues and organs. For example, Neisseria meningitides, commonly present in the nasal passages of about 10 percent of healthy adults, is one cause of bacterial meningitis. This pathogen gains entry into the blood stream by acting through type IV pili on bacterial surfaces, interacting with molecules on endothelial cells to disrupt the tight packing and occasionally escape the mucosal barrier to enter the bloodstream and pass into the meninges of the brain and its surrounding membranes to cause disease and breach the blood–brain barrier [11].

The archetypal leaky syndrome is celiac disease, which is a highly variable complex multi-organ disorder with extra-intestinal complications that are reported to include cortical hyper-excitability, and neurological and motor cortex involvement [12]. Celiac disease can involve many cognitive processes and symptoms, including cerebellar ataxia; peripheral neuropathy; and diverse presentation including seizures, headache, cognitive impairment, and neuropsychiatric diseases. Such processes and symptoms are frequently reported and associated with this disease [13]. These clinical manifestations may be present at disease onset or become evident during course of the disease. Inflammation appears to be central to brain and blood brain barrier involvement.

## 2. The Blood-Immune Privileged Organ Barriers

To understand a possible connection between leaky gut and possible leaky brain, we first examine the barriers under normal physiological conditions. A key component for the brain is the neurovasculature, which limits blood brain barrier permeability and prevents transport of large molecules, many small molecules, and bacteria from entering into the brain. Passage though the barriers and into the lipid-rich brain requires lipid solubility and is accomplished though a diverse transport system. These permeability barriers are highly selective and charged, which essentially separates the circulating blood from the extracellular fluids of the privileged organs and systems, such as the central nervous system. This is basically accomplished through endothelial cells of the capillary wall and acts with other cells like astrocytes with their dendritic feet to ensheath the capillary and associated pericytes to embed in the capillary basement membrane [14].

Inflammation disrupts BBB. Many diseases and physiological stressors that affect the CNS also alter the functional integrity of the BBB [15,16]. They affect the barrier abilities to selectively restrict passage of substances from the blood to the brain. To add to this, hypoxia and/or inflammation and inflammatory process alter the permeability properties and contribute to the pathophysiology of CNS diseases, leading to altered delivery of therapeutic agents to the brain [10]. When we look at what is known with regard to BBB disruption following a hypoxic or inflammatory insult in vivo, the altering of tight junction components at the BBB and potential mechanisms involved in are also highlighted. Detailed understanding of the mediators involved in changing BBB functional integrity in response to hypoxia or inflammatory pain could potentially lead to new treatments for CNS diseases with hypoxic or inflammatory components. Additionally, greater insight into the mechanisms involved in tight junction rearrangement at the BBB may lead to novel strategies to pharmacologically increase delivery of drugs to the CNS.

Recently, a few studies have suggested that the microbiota can directly affect the brain and gut in several ways, including contributing to BBB formation in embryonic development [17]. In that regard, amniotic fluid is not sterile [18] and the development of the BBB and any microbial engraftment begins early in development and continues after birth [17]. Moreover, the amniotic fluid is not sterile and bacterial presence is associated with a disease state [19], but appears vital in immunologic and barrier development. At birth, the mode of delivery may help determine early colonization patterns [1]. Suffice to say, vital organs and biologic systems have developed barriers to infection and passive diffusion of solutes and proteins to host tissues for the host’s survival. The notable barriers are the blood brain barrier, blood gastrointestinal barrier (GBB), blood ocular and blood retinal barriers, blood placenta and blood testis barriers, the blood thymus barrier, and the blood–lung or airway barrier. Each of these barriers protects vulnerable and sensitive organs and systems.

Selective permeability is important and accomplished though tight junctions, composed of endothelial cells and smaller subunits anchored into the endothelium together with transmembrane proteins, such as junctional adhesion molecule, occludins, adherens, and claudins, for example. The junctional proteins in the brain are similar to those of the small intestine. Tight junctions help protect the brain from toxins, chemicals, and pathogens that might be circulating in the bloodstream. Together with selective transport proteins, the barriers allow nutrients, oxygen, amino acids, some drugs, and glucose to enter the cerebrospinal fluid and prevent hydrophobic molecules from passing into the the interfaces of blood–cerebrospinal fluid barriers, namely CSF and choroid plexus. At the same time, it allows the diffusion of many small polar molecules, dissolved gasses, hormones, and hydrophilic molecules. Cells of these barriers actively transport small molecules and metabolic products, such as glucose, mostly using several specific transport proteins. As the barriers share common proteins and features, there is no doubt they may be susceptible to similar mechanisms of compromise or breach, either biochemically or physically. This fact underlies one basis for a plausible leaky gut leaky brain syndrome.

In the gut, the barrier between the body and a lumenal environment is formed by gastrointestinal mucosa, buffering nutrients, microorganisms, and toxins. The barriers are semipermeable, thus allowing efficient transport of nutrients across the epithelium, while excluding entry of potentially harmful small molecules and organisms. The exclusionary properties of the gastric and intestinal mucosa are referred to as the gastrointestinal blood barrier [20].

## 3. Regulating Permeability Barriers

A functional blood–brain barrier is essential to maintaining central nervous system homeostasis. So permeability is essential to restrict movement of substances from the systemic circulation to the brain or other organs, which buffers organs from rapid changes in ionic or metabolic conditions. Limited BBB permeability also protects the brain from exposure to molecules that may be harmless to peripheral organs, but quite toxic to vulnerable neurons in the hippocampus and elsewhere. BBB weakening may be a result of a disturbance in the endothelial cells due to P-glycoprotein dysfunction [21]. In the brain, this can occur with endothelial cells and astrocytes and involve inflammation [22]. Neurons influence permeability in the brain, as does the extracellular matrix and non-neuronal cells including astrocytes, pericytes, and vascular endothelial cells, and for barrier integrity, maintenance of an epithelium is critical. If toxins or microorganisms breach the epithelium, they have unrestricted access to the the systemic circulation. Epithelial integrity also requires balance between cell proliferation and cell death.

In the gut, the alimentary canal is lined by epithelial cells that form the mucosa and, with few exceptions, the gastrointestinal epithelium is tied contiguously through tight junctions, where diversity among epithelial cells affect specific barrier functions. Gastric mucus and mucin, part of the extrinsic barrier consisting of secretions, also affect the barrier by slowing diffusion rates of hydrophilic molecules and aqueous solutions like gastric acid. When the GBB is breached, there are differences in localization of bacterial species. In that regard, it may be axiomatic, but mechanisms of dissemination are varied, whereas commensal bacteria deposit in the lymphatics are not found in the blood stream when a breach occurs, but species like Salmonella, which can establish infections in the blood, liver, or other organs, is by definition pathogenic and patently so and understood to be deleterious in differentiating pathogenic from commensalate forms of microbes. We have evolved with commensals and not pathogens through colonization resistance and other mechanisms, which is one reason why commensal bacteria end up in the nearby lymph nodes and are not found in distal organs in spite of being proximal to the epithelial linings. This is not to say that resident pathogenic bacteria in the gut do not offer beneficial metabolism or assist with co-metabolism in a non-deleterious way.

## 4. Leaky Gut and Celiac Disease

Food sensitivity and emerging glycotoxins have been linked to inflammation [23] and some individuals react to food and dietary proteins as pathogenic or antigenic, causing inflammation of the mucosal barrier and cytokine stress. The inflammasome, comprised largely of cytokines, interleukins (IL)-8, and the pro-inflammatory cytokines tumor necrosis factor (TNF)-α and interferon (IFN)-γ, may play a role in this process and inflammation can induce changes in the expression and/or localization of tight junction proteins, and affect CNS drug uptake [10]. Metabolites of the gut–brain axis are targets for potential drug treatment and drug design [1].

A key point to follow is what happens when the integrity of the intestinal blood barrier is compromised. It has the potential to affect a host of other organ systems, which can be problematic as is patent in celiac disease. Celiac disease is an autoimmune disease, characterized by inflammation of the intestinal mucosa in the small intestine due to an immune response and the loss of tolerance to dietary gluten and gliadin proteins found in wheat, barley, and rye, but its prevalence is actually low globally with prevalence in children ranging from 0.31% to 0.9%, and approximately 1–2% in European adults and 0.4–0.95% in the USA, according to reports by Rimarova [24]. While the occurrence rate of celiac disease is increasing, estimates are more than 2 million people in the United States have the disease, which is 1 in 133 people, and among first-degree relatives of affected individuals, can increase to upwards of 1 in 22 that may have the disease [25]. These numbers do not fully take into account so-called ‘gluten sensitivities’. Although diagnostic methods have advanced in the last decade and improved screening may play a part in the observed increase, what we do not know is how environmental factors or genetics lead to increased prevalence of genetically predisposed individuals [26,27]. Suffice to say, intraepithelial T cell mediated lymphocyte immune recruitment and an autoimmune response can destroy intestinal epithelial cells, which may lead to villous atrophy [28].

Leaky gut may be one of the underlying causes in illnesses involving concomitant downstream disruptions in the blood brain barrier [4,6,10,13,15,16,29] and numerous studies indicate that hypoxia and/or inflammation increase BBB paracellular permeability [12]. We can assume this position from a hypothesis based on neurophysiology, that is, electroencephalography and evoked potentials, which may help uncouple mechanisms involving the gut and brain in celiac disease [13], from the “hyper-excitable celiac brain”, which partially reverts back after a long-term gluten restriction [11]. In addition, arguments are further supported from transcranial magnetic stimulation (TMS), a non-invasive brain stimulation technique used to assess and monitor patients with celiac disease [8]. Taken together, the evidence examined seems to converge on an overall profile of a “hyper-excitable celiac brain”, which these researchers show is gluten-related. Further, excitatory and inhibitory synaptic excitability is observed in patients with celiac disease and TMS suggests there is a subclinical involvement of GABAnergic and glutamatergic neurotransmission [12]. This was even found when celiac patients were asymptomatic, and suggests the brain and its excitation state, and perhaps permeability barriers, could be commonly affected through inflammation in this disease.

The most likely mechanism is mediated though inflammatory processes and even direct bacterial involvement. If bacteria enter the body, it may cause an infection. The intestinal barrier is in continuous contact and co-metabolizes with up to ten trillion microorganisms [2] and represents a major potential portal entry for microbes into the host. Other routes for bacteria to breach a barrier may be through the airway, which could lead to bacterial pneumonia. Celiac disease seems to promote breaches distal to the gut though largely unknown mechanisms [30,31]. In that regard, a Swedish study compared hospitalized individuals with those individuals with celiac disease and found the latter to be at increased risk of sepsis (*p* < 0.001) and offered potential explanations, such as increased mucosal permeability and hyposplenism and altered composition of the intestinal glycocalyx in celiac disease [31].

How digestion-resistant Gliadin protein and peptides (A-gliadin P31–43) can induce a stress response or trigger innate immune responses may be one mechanism in the loss of tolerance to gluten [32]. The innate immune response has been shown to increase zonulin, which is a modulator of intercellular tight junctions and trafficking of macromolecules important in tolerance and immune responses. Dysregulation of the zonulin pathway can increase the permeability of tight junctions and render individuals susceptible to possible autoimmune disorders, cancer, and inflammation [33]. Aging may also affect the permeability barriers as innate immune responses were found to increase in old monkeys consistent with higher microbial translocation burdens, but microbiome profiles from 16S RNA did not show large differences by age. Dietary mechanisms from monkey studies demonstrate gastrointestinal barrier dysfunction and age-related leaky gut and altered responses to a western diet, without celiac disease [7].

When one or multiple host barriers are breached, as in the crossing by a disease causative agent, a critical event in systemic infection occurs and two nonexclusive scenarios can occur. One is the barrier function of a tissue, which can be compromised by physical means or by a host condition that disrupts the integrity of tissues like inflammation or a dysfunction of a host gene product that is implicated in barrier function can occur [30]. Downstream barrier damage can allow direct microbial invasion, or through direct action of microbial gene products. Here, direct breach can mediate microbial adhesion and translocation across host barriers [30].

## 5. Oxidative Stress in Barrier Breach

Redox balance is important for both the brain and the gut and bacteria, and immune cells can modulate oxidative stress, which is involved in the breakdown of the blood brain barrier or gastrointestinal blood barrier. These permeability barriers can be breached and disrupted through several mechanisms like ischemia and reperfusion injury, where infection, bacteria, viruses, fungi, and various systemic diseases act locally in a non-occlusive way, leading to circulatory shock, sepsis, or cardiac insufficiency. Conversely, occlusive ischemia, which refers to direct disruption of blood flow such as during thromboembolism or strangulation, can occur with the mucosa or bowel. Damage actually occurs though reperfusion and production of reactive oxygen or nitrogen species and free radical formation and downstream cascade or bystander effect on brain or mucosal integrity. This can be mediated though peroxynitrite formation, nitrosamines or superoxide anion, and hydroxyl radical, among other means. Any of these stressors can lead to downstream inflammatory process and neutrophil recruitment or a wound healing response, and support for this mechanism is evident through anti-oxidant use like alpha-lipoic acid [34], which may help stabilize a weakening blood–brain or other barrier [35].

Other stressors, such as glycoxidative stress, diabetes, prolonged hyperglycemia, and obesity, are risk factors for gut–blood barrier disruption. To resolve these processes, restoration of epithelium must happen, which can be rapid and is accomplished by a process called restitution. Here, epithelial cells migrate over exposed damaged basement membrane by recognizing exposed proteins. In the small intestine, villi reduce the area of resolution and epithelial proliferation is not involved, which must eventually occur for resolution to restore the normal cellular architecture and function [36]. Advanced glycation end-products and crosslinking in diabetic complications and with aging may also be a mechanism for barrier protein damage with advanced age [37] and could be mediated though glycotoxins from food.

## 6. Cytokine Stress in Barrier Breach

Bacteria and derived lipopolysaccharides or bacterial CpG motifs are highly inflammatory. Understanding the role of innate immunity in the inductive and effector phases of celiac disease are key to understanding the disease in its entirety. The inflammasome activates an inflammatory response and consists of a multi-protein, oligomer assembly complex that promotes the release of pro-inflammatory cytokines, interleukin 1β (IL-1β), interleukin 18 (IL-18), and a myeloid component of the innate immune system. The innate immune system in microbial management includes cytokines, interleukins (IL), recruitment of neutrophils and leucocytes to sites of infection, complement cascade activation, and antibody complexes. This is in contrast to the adaptive immune system, antigen presentation, and T-cell or cell-mediated immune responses. Together, the gut–blood and blood brain barriers provide a diffusion selective barrier that helps monitor the entry of substances and bacteria into host organs and eventually the brain [11,15,38], and also monitors movement of T-cells across these barriers, without extravasation and helping regulate the inflammatory cascade.

Other early response cytokines to host microbial challenges include TNF and IL-1. The proinflammatory cytokines (IL-6) are acute phase cytokines, which also stimulate B-cell proliferation and are produced and amplified by T-cells, monocytes, macrophages, fibroblasts, and endothelial cells. Conversely, inhibitory cytokines like IL-10 act in opposing fashion including inhibiting production of IL-12, co-stimulator molecules, and major histo-compatability-II molecules. IL-15 stimulates natural killer cell and T8 memory T-lymphocyte (T-cells) proliferation. Macrophages and other cells produce IL-15 and IL-18, which stimulate the production of interferon-gamma by natural killer cells and T-lymphocytes, which induce cell-mediated immunity. Further, interferon-alpha can modulate the microbiota and innate processes such as polarization of dendritic cells and, along with Toll-like receptors (TLR) such as TLR9, recognize bacterial CpG repeat motifs [39]. These are an integral part of the innate immune system. Conversely, serologically positive celiac cases can present with normal mucosa and further complicate the leaky gut picture [40].

Inflammation in the body may lead to direct and even disruptive effects on the brain via the blood–brain barrier. During systemic inflammation, whether in the form of infection, the BBB may undergo changes that may cause the BBB in patients with neurological disease to be abnormally sensitive to the effects of systemic inflammation [41]. Leukocyte infiltration in celiac disease and changes in tissue architecture of the small intestine have been found in genetically susceptible individuals [42]. Clinical manifestations and the histological changes in patients with celiac disease, including changes in regulatory T cells, called Tregs, are not well understood but are implicated in the loss of gluten tolerance. The effector T-cell response has been characterized and after oral wheat challenge, significant numbers of circulating gluten-specific Tregs have been found and effector T cells have been shown to increase in celiac disease. Tregs reside within the pool of memory T cells and, in celiac disease, exhibit significantly reduced suppressive functions. This can normalize when gluten is eliminated from the diet. Studies have shown the gluten-specific FOXP31CD391 are involved in the disease and CD41 T cells may involve suppressive function, as can Treg cell dysfunction, which can contribute to disease pathogenesis [32].

## 7. Brain Disorders and the Microbiota

A dysfunction of the blood brain barrier leading to a ‘leaky brain’ can be linked to various neurological diseases, including autistic spectrum disorder (ASD) [43], dementia, Alzheimer’s disease, depression, and schizophrenia [18,44]. A breakdown in the blood brain barrier was observed in patients with major psychiatric illnesses [45]. Moreover, the blood–brain barrier may become ‘leaky’ in select neurological diseases that have an immunologic component, such as multiple sclerosis (MS) [46,47], Alzheimer’s disease, brain trauma, edema, brain cancers [48], amyotrophic lateral sclerosis, meningitis [11], and systemic diseases such as liver failure [49]. Moreover, co-metabolism within the gut–brain–endocrine interactome play a role in the same neurodegenerative disorders, including Parkinson’s disease (PD) and even autism, appear to have a microbial-driven component in their pathogenesis [50]. Moreover, known microbes are implicated in contributing to the susceptibility and pathogenesis of these disease and BBB permeability and disruption has been established in Alzheimer’s disease (AD), which may allow peripheral blood, amyloid beta, and cytokines to enter the brain and contribute to pathogenesis in vulnerable neurons [51,52].

Recently, we describe how microbiomes of the gastrointestinal tract play important roles in controlling very integral segments of our neurobiology, mental, behavioral, and even overall health, including memory, depression, mood disruption, and anxiety [2]. Abnormal behavior and cognition are found together with dysbiosis or the so-called pathobiont overgrowth syndrome, which can be one cause or consequence of leaky gut and loss of the intestinal blood barrier [2] and a disrupted intestinal barrier may have an abnormal microbiome, especially if antibiotics are involved. Further, Collins and colleagues found changes in both brain chemistry and behavior regarding the microbiota [1].

Antibiotic use and depression and psychiatric comorbidities occurred in irritable bowel disease and have been associated with a systemic inflammation [15]. Inflammatory reactions can disrupt the blood brain barrier, the so-called leaky brain, leading to increased CSF protein and their translocation. Inflammation promoting substances, like lipopolysaccharide, may pass into the brain. The BBB displays a paucity of pinocytosis, but inflamed meninges can disrupt the barrier, which becomes more permeable during inflammation and can permit substances like antibiotics and phagocytes to move across the BBB [53].

Stress has immunologic consequences as well, and also has a role in these interactions. For example, IL-1 and IL-6 are able to increase cortisol release by the stimulation of hypothalamic pituitary arm of the microbiota gut brain (MGB) axis and stressed or depressed patients often exhibit perturbations in this axis, resulting in elevated cortisol levels. In addition to the microbiome, the mycobiome participates in modulating cytokine production, with IL-6 [54]. Thus, cytokines produced at the gut reach the brain via the bloodstream, where they affect immune pathways. In autism, underprovided most notably in the hypothalamus and circumventricular organs, it was found some molecules may cross the blood–brain barrier and modulate brain area stimulation [55].

Microbial interactions with the BBB may involve crossing of brain microvascular endothelial cells in a vacuole via transcytosis or through the intercellular junctional spaces via paracytosis, or via the so-called Trojan horse scenario, while inside a host cell as a means to cross the barrier [29]. The microbiota gut brain (MGB) axis can indirectly affect the brain as a function of the network of metabolic communication signaling together with other molecules moving between the gut and the brain and between host metabolism and pathogenic or commensal resident gastrointestinal bacterial metabolism. They act to modulate both the gastrointestinal tract and the central nervous system [2,6,10]. Ultimately, the intestinal microbiota can communicate with the brain via these axes to influence aspects of brain development and behavior and influence a broad spectrum of diseases [2]. For example, we established there was significance for trace amines in the etiology of neuropsychiatric disorders, as we reported in the literature [1], which are chemically related to the catecholamines and 5-hydroxytryptamine (5HT) in the CNS, and bacteria can produce 5-hydroxyindoleacetic acid (5HIAA), homovanillic acid (HVA), serotonin and 5HT which can be traced to the CNS [2]. The MGB axis and its metabolite cross-talk, referred to collectively as the biochemical co-metabolism that occurs between the host and the microbiota, is not yet characterized in any ‘leaky’ syndrome. The construct of ‘leakiness’ within the gut–blood and blood–brain barriers may be affected by microbes as elucidated. One may extend the construct of ‘leakiness’ to include microbial metabolites and exogenous compounds that pass system barriers.

When we consider epigenetic modifications in ASD individuals, we find post-translational modification like DNA methylation, histone modification, microRNA dysregulation, and gene polymorphisms [56,57]. Epistasis is an important interaction with diet and likely contributes to ASD pathobiology, where changes in gene expression, indicating alterations in common molecular pathways including the immune system, are involved [58]. Maintaining healthy brain function and barriers are vital, as these claims suggest. In ASD, increased cytokine levels of IL-beta, IL-6, IL-8, and IL-12p40, among others, were associated with ASD impairments in stereotypical behaviors and in regression, suggesting that dysfunctional immune responses could affect core behaviors in ASD [5]. However, there has been no evidence of albumin or immunoglobulins found in the brains of ASD individuals and many groups are currently exploring these and other markers in Alzheimer’s disease (AD), schizophrenia, and other psychiatric disorders [59].

Support of the notion of bacteria helping preserve barriers is evident in germ-free animal experiments, where an increased BBB permeability has been shown, coming from a reduction in tight junction protein expression. We show that changes in tight junctions can be improved after microbial colonization of rodent digestive tract with short chain fatty acid producing bacteria or after short-chain fatty acids administration or supplementation after *C. difficle* infection [60]. Conversely, probiotics could potentially help prevent leaky gut or the consequences of symptoms and possibly restore colonization resistance to the species contributing to ‘leakiness’ [61]. Similar to our work with the short chain fatty acid butyrate, Hoyles and colleagues demonstrated that propionate had protective effects on the BBB by inhibiting pathways associated with non-specific microbial infections via a CD14-dependent mechanism [62]. They show that suppressed expression of LRP-1 and NRF2 (NFE2L2) signaling protected the BBB from oxidative stress, which can affect BBB barrier integrity.

Finally, one important growing brain disorder is ASD, which affects up to 1 in every 68 children born in the united states [43]. The one study that may best support the leaky gut, leaky brain hypothesis comes from a post-mortem, nonconventional study that compared cerebral cortex and cerebellum from 8 ASD individuals and 33 age-matched controls according to mode of death and preagonal conditions [16]. This group examined gene expression related to blood–brain barrier integrity and inflammation and found alterations. They then analyzed gastrointestinal tissue from a different cohort of 9 postmortem individuals with ASD and 12 controls. They found that 75 percent of the ASD individuals had reduced barrier forming components and 66 percent showed increased expression of intestinal permeability molecules. A similar approach from the same research group examined 10 schizophrenia brains and 15 healthy controls and found gene expression for schizophrenia was similar to the ASD study and protein expression appeared to follow a trend similar to ASD, namely, a trend toward reduced CLDN-5, CLDN-3, and reduced cortical CLDN-12 protein levels associated with blood–brain barrier integrity and function. They also found that increased proinflammatory cytokines were also affected in the disease, which supports the hypothesis that neuroinflammation contributes to both diseases and can affect barrier permeability [16]. These results allow us to draw conclusions up to causality between loss of barrier function and neurobehavioral changes, which is compatible with ASD.

Regardless of whether or not the previous study design led to sufficient bias and was controversial, it does add fodder to a growing body of evidence that the blood brain barrier in autism is disrupted and is related to gastrointestinal barrier or breach. We have suggested neuroinflammation and oxidative stress occurs in individuals affected with ASD in mechanistic studies [43]. What is still needed is deep sequencing of microorganisms in the gut of ASD patients to determine which microbial organisms are most perturbed in ASD and whether this dysbiosis occurs at prodromal stages. We have suggested that probiotic or synbiotic treatment might be able correct or prevent disease pathogenesis or even lessen the behavioral issues that affect this spectrum of diseases.

When considering dietary aspects of these net interactions, the reader must understand that the new concepts represent novel pathways and divergent views from dogma and classic understanding, which builds on our previous work of the metabolic interaction between any host and any given organismat the small molecule level [19]. Indeed, our diet, lifestyle, and medications, particularly antibiotics, influence and shape the gut microbiome throughout our life. The converse is also true. The interaction is always in flux and the result is net metabolite or end-product production, with positive and negative effects on human health.

## 8. Conclusions

The gut certainly can influence the blood brain barrier through gastro-intestinal-derived hormonal secretion, allowing some drugs, amino acids, and small molecules to permeate barrier systems and even influence cytokine production, which is one inflammatory piece of the innate immune system issue. Bacteria can pass the human permeability and blood barriers. While this is evidence that bacteria can affect distal organs, there are some unfortunate issues in the best found studies one can use to argue for a leaky gut/leaky brain. Nevertheless, we cannot preclude that there is not something here and we should explore this idea further. Eventually, it may be possible to use the metabolism from commensals to either affect human health positively or limit negative effects from disease. We anticipate developing microbial probiotics or symbiotics that could offer new treatments to include strengthening the gut, the brain, or related permeability barriers.

The future outlook and clinical perspectives on exploring translational application for anaerobic bacteria administration as a treatment approach is promising. Developing and characterizing the microbiome holds tremendous promise for science and drug treatments. It is not hype but incredibly promising, even revolutionary, to look the the microbiota for improving our metabolism and ultimately humanity. We highlight the potential translational role for the use of commensal microbes as drugs and for general clinical practice to prevent or treat disease. We should support further study and research funding, as this approach would provide societal benefit and possibly better treatments or cures. Unfortunately, only a small number of studies suggest the microbiota can directly affect the BBB, and concomitantly the GBB [8]. Further, oxidative stress and inflammation are simple jargon-loaded words we use to describe very complex mechanisms, such as superoxide oxidative burst or cytokine stress, which can be mediated though the microbiome. Nevertheless, the possibility of multiple leaky syndromes and mechanisms may be involved in disease pathogenesis.

One short-coming of these studies for a leaky gut/leaky brain is that they fail to offer detailed explanations for the findings or a unifying construct, something more than anecdotal or post-mortem proof that a physiologic effect is unequivocal. I like to say that when it comes to opining on fringe concepts in medicine or complementary alternative theories in practice, ‘I like to keep an open mind. However, not so open a mind that my brains fall out’ or, in this case, leak out. Regardless of where one falls on the continuum, there is just not enough evidence to suggest that one should eliminate gluten from the diet or eat gluten-free, or that everyone should stop eating gluten-containing foods, especially when they taste so good. When there is such compelling evidence, I will join the ranks of the proponents on the proverbial soap box and proclaim it so. Until that time, the jury of science is rendering a verdict on gluten and calling for more funding to explore the underpinnings of leaky gut, leaky brain.
